# Prevalence and Intensity of Soil-Transmitted Helminth Infection among Rural Community of Southwest Ethiopia: A Community-Based Study

**DOI:** 10.1155/2019/3687873

**Published:** 2019-12-14

**Authors:** Eyob Tekalign, Mitiku Bajiro, Mio Ayana, Abebaw Tiruneh, Tariku Belay

**Affiliations:** ^1^College of Health Sciences, Department of Medical Laboratory Sciences, Mizan-Tepi university, Mizan, Ethiopia; ^2^Institute of Health, School of Medical Laboratory Sciences, Jimma University, Jimma, Ethiopia; ^3^Molecular Biology Research Center, Jimma University, Jimma, Ethiopia

## Abstract

**Background:**

Helminths are significant contributors to global health problems. Subgroup soil-transmitted helminths are among the listed neglected tropical diseases. The rural inhabitants often suffer from heavy infection, particularly children and pregnant women.

**Objective:**

The study aimed at determining the magnitude and intensity of soil-transmitted helminth infection and associated risk factors in the study area where the prevalence and intensity of the infection are yet unknown at the community level.

**Method:**

A community-based cross-sectional study was conducted between April and June 2016 on 377 individuals. Systematic random sampling was utilized to select the households. Lottery method was used for study subject selection in the households. Sociodemographic and risk factor data were collected using a pretested questionnaire. Parasitological tests were processed using Kato-Katz thick smear and duplicate direct wet mount analysis of the stool sample.

**Results:**

A total of 377 subjects aged from 2 to 55 years were enrolled in the study, of which 211 were female (56%) and 166 were male (44%). The overall prevalence of soil-transmitted helminths was 265 (70.3%). The females shared more (36.6%, 138) as compared to males (33.7%, 127) at *P* < 0.05. Of all identified soil-transmitted helminths, *Trichuris trichiura* was the predominant infectious agent (66.8%, 252) followed by *Ascaris lumbricoides* (16.4%, 62) and hookworm (14.1%, 53). Gender (AOR: 1.67 (95% CI: 1.034–2.706)), lack of fruit washing before consumption (AOR: 1.7 (95% CI: 1.1–2.6)), open defecation habit (AOR: 1.75 (95% CI: 0.921–3.338)), and drinking untreated water (AOR: 1.994 (95% CI: 1.019–3.90)) were significantly associated with soil-transmitted helminth infection.

**Conclusion:**

High prevalence of STH infection was still an important health issue of the community even after the implementation of the health extension program. Hence, intervention considering all population of the residents as eligible to deworm and health education are mandatory.

## 1. Introduction

Helminths are significant contributors to global health problems [1, 2]. Soil-transmitted helminths (STHs), a subgroup within helminths transmitted through contaminated soil and water with faecal matter, are the leading neglected tropical diseases among the 20 diseases on the WHO list. The infection persists in humans where behavioral, environmental, and socioeconomic factors are linked to the distribution. The rural inhabitants often suffer from the infection particularly preschool school-aged children and pregnant women [3, 4].

Morbidity is related to heavy infection intensity. In addition, the infection is associated with malabsorption, anemia, and reduced physical and mental growth in childhood. Globally, an estimated 5.3 billion people are living in areas of stable transmission of *Ascaris lumbricoides*, *Trichuris trichiura*, *Necator americanus*, and *Ancylostoma duodenale*. School-age children account for 1 billion [5]. Current estimate suggests that about 2 billion people are infected with STHs [6, 7]. Sub-Saharan Africa, Southeast Asia, China, India, and South America account for the highest burden [8, 9].

Ethiopia is among sub-Saharan African countries with the highest burden and where the disease remains an important public health problem [10]. To empower households and rural communities to promote health and prevent parasitic disease, the country launched health extension program. The program creates model families who adequately adopt the desired health practices of hygiene and environmental sanitation and disseminate health information to other community members [11, 12]. Tangible improvements in hygiene and sanitation coverage assumed the reduction of the parasitic and other communicable diseases through fixed and outreach-based regular activities of health extension workers [13]. Though the implemented program has brought tangible improvement, no data were available on the epidemiological distribution of STH. Hence, to determine the magnitude, intensity, and associated factors of the major soil-transmitted helminths at the community level appears very much necessary to focalize areas for integrated interventions.

## 2. Materials and Methods

### 2.1. Study Area and Population

The study area is located in Southern Nations, Nationalities, and Peoples' Regional State (SNNPR), Ethiopia. SNNPR is one of the nine regional states in Ethiopia and consists of 14 zones and 5 special districts. Bench Maji Zone is one of the aforementioned zones and its administrative center is Mizan-Teferi. It is located at 575 km from Addis Ababa. Zemika Kebele (smallest administrative unit) where this study was conducted is found in Bench Maji Zone and home to 5246 inhabitants with about 1048 households. Of the total households, 200 households were model families. The study area is characterized by “Woyena dega” type of climatic condition, lying at an altitude ranging from 1300 to 1750 m above sea level with an annual average rainfall of 1400 mm^3^ and temperature ranging from 15°C to 27°C with an average of 20°C.

There were 14 villages in the kebele; eight of which, Mashela, Kisin, Ketembus, Kaykin, Selam, Yalin, Yakin, and Tabya, were randomly selected to be included in this study. A total of 561 households were available in the selected kebeles.

### 2.2. Study Design and Study Period

A community-based cross-sectional study was implemented to estimate the prevalence, intensity, and associated factors that predispose the community for getting soil-transmitted helminths. The study was conducted from April to June 2016.

### 2.3. Sample Size and Study Population

The sample size was determined using a single population proportion formula [14] taking the maximum proportion (50%) because of the absence of recent data of STH at the community level considering 95% confidence level (*z*=1.96), 5% marginal error. Thus, the calculated sample size was 384. Correction had been done to get a true sample size as the population <10,000, and considering a 5% nonresponse rate, the true sample size of 377 study subjects were randomly selected from 75 households after proportionally allocated to each selected village. All residents in the study area were considered as a target population and constituted the study population. Participants' age ≥2 years from the selected village were included in the study. Individuals who had treatment for intestinal parasites in the last three months prior to the survey, who had a chronic disease, and who were not able to submit a stool sample were excluded.

### 2.4. Data Collection

#### 2.4.1. Questionnaire Survey

The questionnaire was pretested in communities with similar characteristics for the necessary adjustment to be made. Structured questionnaires were prepared in English and translated to Amharic to distribute to trained data collectors. Sociodemographic characteristics and risk factors that predispose to STH infection were collected using the questionnaire.

#### 2.4.2. Stool Collection and Processing

After completing the questionnaire survey, the individuals who agreed to participate in the study were provided with marked clean labeled plastic stool cups and instructed to bring approximately 5 grams of their own stool. The samples were examined for detection and identification of intestinal parasite at field setting by two laboratory technologists using double direct saline techniques within 30 minutes of collection. After the completion of the direct stool examination, aliquot of each sample was transported to a regional public health laboratory within the same day using a cold box for the quantitative examination of *Ascaris lumbricoides*, *Trichuris trichiura*, and hookworms using Kato-Katz technique [15]. The number of eggs counted was multiplied by 24 to get egg per gram in stool (EPG). Infection intensity was categorized as light, moderate, and heavy infection for common STH infections following the standard procedure of the WHO [16].

### 2.5. Quality Control

The questionnaire and other materials were pretested before the actual data collection. Data collectors were trained on how to conduct the interview and how to collect the stool samples. Laboratory examinations were performed by experienced medical laboratory technologists. Stool samples were randomly selected for quality control and examined by a third person who was blinded for the previous test result.

### 2.6. Data Analysis

The data were entered into MS excel, cleaned, and imported to SPSS version 20 for statistical analysis. Descriptive analysis including frequency, mean, and range was used to summarize demographic characteristics of the study participants. The average EPG was calculated using geometric mean (GM). Intensity level was graded according to the WHO standard [17]. Bivariable analysis was computed for the association of each independent variable with the dependent variable. Candidate variables for multivariable analysis were selected based on *P* value result during bivariable analysis (*P* < 0.2). *P* values <0.05 were considered as statistically significant.

### 2.7. Results

#### 2.7.1. Characteristics of the Study Participants

Three hundred seventy-seven subjects were enrolled in the study (response rate 100%). The age was ranged from 2 to 55 with the median age of 13.0 ± 11.8 years. Children ≤14 years took the highest percentage. Females were more than half (56%, *n*=211) as compared to male counterparts (44%, 166). Out of the total, 84 (22.3%) were from model families. As the finding profile, 334 (88.3%) had their own latrine in their house. However, most of them (83.4%, 315) had open field defecation habit. Likewise, for majorities (66.3%), drinking water supply was from unprotected sources. Habit of always washing hands before meal was practiced in 351 (93.1%) subjects (Table 1).

#### 2.7.2. Prevalence and Intensity of Soil-Transmitted Helminths

**Figure 1:**
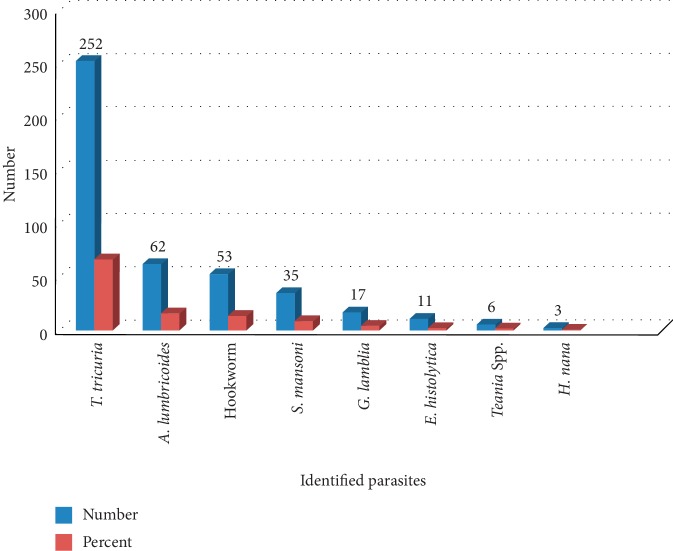
Identified parasitic species among the study participants in Zemika Kebele, 2016.

The overall prevalence of intestinal parasites was 274 (72.7%). Of these, soil-transmitted helminth infection took 265 (70.3%). The females shared more (36.6%, 138) as compared to males (33.7%, 127). Infection among model families was more than half (59.5%) (Table 2). Regarding infection intensity, *Trichuris trichiura* and *Ascaris lumbricoides* had light and moderate infection intensity; however, heavy infection of *Trichuris trichiura* was found in four participants. Light infection of hookworm was also documented. The overall intensity calculated using geometric mean showed light infection of STH (Table 3). Species-specific report of the study revealed that eight species were identified. *Trichuris trichiura*, *A. lumbricoides*, and the hookworms comprised 66.8%, 16.4%, and 14.1%, respectively (Figure 1). Infection of a single host with multiple parasites was recorded in the study area. Among those study participants who had complete parasitological data, 44.2% of them were the host for multiple parasites (Table 4).

#### 2.7.3. Factors Associated with Soil-Transmitted Helminths

Logistic regression was analyzed whether the overall STH infection was significantly associated or not with the potential risk factors. In the study, sex, lack of fruit washing before consumption, open defecation habit, and drinking untreated water were identified as risk factors. Based on the analysis, males were 1.67 times more often get infection than females (AOR: 1.67 (95% CI: 1.0–2.7)), and study subjects who had no practices of washing fruit (AOR: 0.46 (95% CI: 0.27–0.79)) were more likely to acquire the infection. Open defecation (AOR: 1.75 (CI: 0.9–3.3)) and drinking untreated water (AOR: 1.9 (95% CI: 1.0–3.9)) were also the identified risk factors. Being a model family or being a nonmodel family was not associated with STH infection (Table 2).

### 2.8. Discussion

Infection with soil-transmitted helminths in the rural community of the study area is still a major problem. The overall STH infections had varied prevalence in different areas. It was evident that the study area had the highest prevalence (70.3%) of soil-transmitted helminths. The finding added further evidence on the problem despite the available safe, effective medicines and the launched health extension program. This finding reported lower STH infection as compared to the earlier report in the neighboring community of Jimma (83%) [18], higher than the two studies reported in Nigeria (44.7% [19] and 63.1% [20]). In Ecuador, the prevalence was almost near to the present study, 65% [21]. The previous study in Shewa Robit Ethiopia reported a little bit higher prevalence (72.7%) than the present study [22]. Soil-transmitted helminths were also common in China rural community in more than half of the participants (51.7%) [23]. Based on the higher prevalence finding of the conduced study, the area is still under the category of high transmission after the implementation of the health extension program [24]. The morbidity of soil-transmitted helminths is associated with the intensity of infection. The current study showed that the overall intensity of STH was under the category of light infection although four individuals showed heavy infection with trichuriasis. The result of this study was not in accordance with the result of the rural Malaysian community in which study trichuriasis and ascariasis were caused by heavy infection among more than half and a quarter of the study participants, respectively [25].

In the current study, multiple infections were common among the study participants. The finding was in accordance with other studies done in Ethiopia and elsewhere [26, 27]. The revealed finding in this study was higher compared to Gilgel Gibe, Ethiopia [28]. However, light infection is not associated with morbidity; polyparasitism among the community may lead to some conditions like anemia [29]. *T. trichiura* was the most predominant among the detected and identified STH infection in this study. In Nigeria [19] and Bushulo, southern Ethiopia [3], and in rural Abaya, Ethiopia [30], a lower prevalence of *T. trichiura* was reported. In Gilgel Gibe dam area of southwest Ethiopia, hookworm was the predominant, but comparable prevalence of *Ascaris lumbricoides* with the present study was reported [31]. There was a difference in the prevalence of STHs among models and nonmodels with no significant association. This may suggest that a significant STH burden reduction is challenging by only training group unless behavioral change monitoring and government intervention activities to be supplemented. The prevalence rate in females was slightly higher than in males with a significant level of association. The finding is dissimilar with the study conducted in Nigeria; males were highly infected [19]. In other studies, sex difference was not significantly associated with STH infection [22]. The obtained sex-specific differences may be related to home activities of women that expose them frequently to the source [32]. Environmental sanitation and personal hygiene are among the main components for the control and elimination of the STHs. Latrine coverage and utilization is the core activity to be improved among the community, and thus, it is the one included in the health extension program packages [33]. Contrary to the high coverage of latrine among the studied community, open field defecation was alarmingly high. The prevalence of STH was significantly higher among those who reported open field defecation compared to those who do not have the practices. The prevalence of STH in the study area was also facilitated by fruit consumption behavior of the community. In the study area, the habit of drinking treated water was poor; however, it reduces the infection with the STHs. In this study, it was evidenced those having poor practice of drinking treated water were twice more likely to get infected with STHs than those having the practice of drinking treated water. Consumption of unwashed fruits also attributed to the high prevalence of STH infection. This study suggested that in those not having the practice of eating washed fruits, the prevalence was higher. Some studies conducted in Ethiopia demonstrated that there is a possibility of fruits can be contaminated with STHs [34, 35].

## 3. Conclusion and Recommendation

The study showed a high prevalence of soil-transmitted helminths. A significant difference in infection was observed among females and males. *Trichuris trichiura* was the major etiological agent. This finding in particular suggests poor practice of drinking treated water, poor practice of eating washed fruits, and open defecation practice among the study subjects were contributed to the high prevalence of STHs. The finding also suggested the need to influence the factors related to the contribution of this high prevalence. Despite the light intensity of infection, the recorded polyparasitism may lead to the infected community to other health consequences. Further baseline survey on knowledge, attitudes, and practice of the community as well as the health extension workers on neglected tropical disease has to be assessed. In conclusion, intervention focusing on the currently identified risk factors and community-based chemotherapy considering all as eligible population with the provision of health education and monitoring the behavioral change of the residents is mandatory. In addition, supplying safe water to the community has to be reminded.

### 3.1. Limitation

In the study, single Kato-Katz was conducted on a single stool sample which can affect the level of intensity.

## Figures and Tables

**Table 1:** Sociodemographic characteristics of Zemika Kebele residents, 2016.

Characteristics	Subjects	Percentage
Age		
≤14	213	56.5
>14	164	43.5
Sex		
Male	166	56
Female	211	44
Study subjects		
Model family	84	22.3
Nonmodel family	293	77.7
Family size		
≤5	218	57.8
>5	159	42.2
Latrine availability		
Yes	334	88.6
No	43	11.4
Drinking water		
Unprotected source	250	66.3
Protected source	127	33.7
Water for domestic		
Unprotected	348	92.3
Protected	29	7.7
Fruit washing before consumption		
Yes	228	60.5
No	149	39.5
Drinking treated water		
Yes	95	25.2
No	282	74.8
Hand washing before meal		
Sometimes	33	8.8
Always	334	91.2
Open defecation		
Yes	315	83.4
No	62	16.6

**Table 2:** Factors associated with soil-transmitted helminth infections among Zemika kebele residents, southwest Ethiopia, 2016.

Variables	STH-positive case (%)	COR (95% CI)	AOR (95% CI)	*P* value
Age				
≤14	151 (70.8%)	Ref.		
>14	114 (69.5%)	0.936 [0.600–1.461]		
Gender				0.036^*∗*^
Female	138 (65.4%)	Ref.	Ref.	
Male	127 (76.5%)	1.723 [1.090–2.722]	1.673 [1.034–2.706]	
Subjects				0. 249
Nonmodel families	215 (73.4%)	Ref.	Ref.	
Model families	50 (59.5%)	0.534 [0.321–0.886]	0.711 [0.397–1.271]	
Family size				
≤5	148 (67.9%)	Ref.		
>5	117 (73.4%)	1.318 [0.838–2.072]		
Latrine				
Not available	234 (70.0%)	Ref.		
Available	31 (73.8%)	0.906 [0.447–1.836]		
Drinking water				0.655
Unprotected	161 (64.4%)	Ref.	Ref.	
Protected	104 (81.9%)	2.500 [1.485–4.207]	1.157 [0.610–2.192]	
Domestic water				0.383
Unprotected	238 (68.3%)	Ref.	Ref.	
Protected	27 (93.1%)	6.239 [1.458–26.706]	2.049 [0.409–10.252]	
Fruit before consumption				0.005^*∗*^
Not washed	180 (78.9%)	Ref.	Ref.	
Washed	85 (57.0%)	0.354 [0.225–0.558]	0.464 [0.271–0.793]	
Drinking treated water				0.044^*∗*^
No	187 (66.3%)	Ref.	Ref.	
Yes	78 (82.1%)	2.331 [1.305–4.162]	1.994 [1.019–3.901]	
Open defecation				0.047^*∗*^
No	34 (54.8%)	Ref.	Ref.	
Yes	231 (73.3%)	2.265 [1.295–3.961]	1.754 [0.921–3.338]	

^*∗*^Significantly associated with STH.

**Table 3:** Intensity of STHs among the Zemika Kebele community, southwest Ethiopia, 2016.

Parasite species	Geometric mean	Intensity of infection		
		Light	Moderate	Heavy
		Number (%)	Number (%)	Number (%)
*Trichuris trichiura*	544 egg/gram	150 (62.2%)	87 (36.1%)	4 (1.1%)
*Ascaris lumbricoides*	2507 egg/gram	41 (70.7%)	17 (29.3%)	0 (%)
Hookworm	162 egg/gram	52 (100%)	0 (%)	0 (%)
*Schistosoma mansoni*	138 egg/gram	13 (39.4%)	20 (60.6%)	0 (%)

**Table 4:** Multiple infections among Zemika Kebele residents, 2016.

Polyparasitism	Gender		
	Female	Male	Total
	Number (%)	Number (%)	Number (%)
Infection with one species	78 (37)	72 (43.4)	150 (39.8)
Infection with two species	48 (22.7)	45 (27.1)	93 (24.7)
Infection with three species	12 (5.7)	7 (4.2)	19 (5.0)
Infection with four species	6 (2.8)	5 (3)	11 (2.9)
Infection with five species	1 (0.5)	0 (0)	1 (0.3)
Total	211	166	377

## Data Availability

All original data used to support the findings of this study are available from the corresponding author upon request.
